# Long COVID elevated MMP-9 and release from microglia by SARS-CoV-2 Spike protein

**DOI:** 10.1515/tnsci-2022-0352

**Published:** 2024-10-13

**Authors:** Duraisamy Kempuraj, Irene Tsilioni, Kristina K. Aenlle, Nancy G. Klimas, Theoharis C. Theoharides

**Affiliations:** Institute for Neuro-Immune Medicine (INIM), Dr. Kiran C. Patel College of Osteopathic Medicine, Nova Southeastern University, Ft. Lauderdale, FL, 33328, United States of America; Miami Veterans Affairs (VA) Geriatric Research Education and Clinical Center (GRECC), Miami Veterans Affairs Healthcare System, Miami, FL, 33125, United States of America; Laboratory of Molecular Immunopharmacology and Drug Discovery, Department of Immunology, Tufts University School of Medicine, Boston, MA, 02111, United States of America

**Keywords:** brain inflammation, long COVID, luteolin, mast cells, microglia, MMP-9

## Abstract

**Objective:**

Long COVID is a major health concern because many patients develop chronic neuropsychiatric symptoms, but the precise pathogenesis is unknown. Matrix metalloproteinase-9 (MMP-9) can disrupt neuronal connectivity and be elevated in patients with long COVID.

**Methods:**

In this study, MMP-9 was measured in the serum of long COVID patients and healthy controls, as well as in the supernatant fluid of cultured human microglia cell line stimulated by recombinant severe acute respiratory syndrome coronavirus 2 Spike protein, as well as lipopolysaccharide (LPS) and neurotensin (NT) used as positive controls. MMP-9 was measured by commercial enzyme-linked immunosorbent assay.

**Results:**

MMP-9 was significantly elevated in the serum of long COVID patients compared to healthy controls. Moreover, there was significant release of MMP-9 from a cultured human microglia cell line stimulated by LPS, NT, or Spike protein. We further show that pretreatment with the flavonoids luteolin and tetramethoxyluteolin (methlut) significantly inhibited the release of MMP-9 stimulated by the Spike protein.

**Conclusion:**

MMP-9 from Spike protein-stimulated microglia could contribute to the development of long COVID and may serve as a target for treatment including the use of luteolin.

## Abbreviations

CDC: Centers for Disease Control and Prevention
ECM: extracellular matrix
ELISA: enzyme-linked immunosorbent assay
LPS: lipopolysaccharide
MMP-9: matrix metalloproteinase-9
NT: neurotensin
SARS-CoV-2: severe acute respiratory syndrome coronavirus 2

## 1 Introduction

Long COVID has been considered the “Next National Health Disaster” in the United States [1]. As many as 50% of those infected with severe acute respiratory syndrome coronavirus 2 (SARS-CoV-2) may develop long COVID [2], especially neuropsychiatric symptoms [3] known as Neuro COVID [4,5] that may last up to 2 years, [6], and maybe more common in patients with other chronic neurologic diseases [7]. However, the precise pathogenesis of long COVID has yet to be fully elucidated [8].

SARS-CoV-2 Spike protein may enter the brain from the nose through the nasal neural mucosa following the olfactory nerve tract [9]. While the exact brain pathogenetic mechanisms remain unclear, evidence points to the involvement of neuroinflammation [10,11], especially perivascular inflammation [12] and blood–brain barrier (BBB) disruption [12,13], leading to neuronal damage [14]. Autopsy studies of patients with COVID-19 showed severe neuronal loss in the capillaries of the choroid plexus [15], as well as neuronal necrosis and glial cell hyperplasia [16]. A 2-year longitudinal study using plasma proteomics to probe long COVID reported that pathways related to neuron generation and differentiation were persistently suppressed [17].

A critical component of neuronal connectivity is the extracellular matrix (ECM) that can be disrupted by matrix metalloproteinases (MMPs). MMPs are important in tissue formation, neuronal network remodeling, and BBB function [18]. Matrix metalloproteinase-9 (MMP-9) has emerged as an important molecule in neuropsychiatric [19,20] and neurodegenerative disorders [21]. MMP-9 can disrupt the polysaccharide scaffolding of the brain matrix and digest tight junction proteins, thus disrupting neuronal connectivity [22]. MMP-9 can cause vascular inflammation and increase BBB permeability [23]. MMP-9 levels were elevated in the serum of COVID-19 patients and were associated with disease severity [24,25].

We investigated serum levels of MMP-9 in long COVID patients, whether recombinant SARS-CoV-2 Spike protein could stimulate the release of MMP-9 from cultured human microglia and whether the flavonoid luteolin could inhibit this process.

## 2 Methods

### 2.1 Patients

Patients (*n* = 13, 6 females and 7 males, mean age was 57 years old) were recruited from Southern Florida. Study participants were recruited from a companion longitudinal study of residents of South Florida who tested positive for SARS-CoV-2. Nova Southeastern University IRB No: 2020-590 (approved January 6, 2021, expires January 11, 2025). Individuals were recruited from those who tested positive for COVID-19 in Broward County and were included in the Florida Department of Health Bureau of Epidemiology COVID-19 surveillance data, or in the records of Community Health of South Florida Inc. (CHI), a Federally Qualified Health Center in Miami-Dade County or in the records of participating community-based provider offices. The inclusion/exclusion criteria for unrecovered individuals were fatigue, as well as one additional symptom that began after positive SARS-CoV-2 test and that the participant self-reported experiencing “a good bit of the time,” “most of the time,” or “all of the time” during the past month. Individuals were 18–65 years old and were able to consent to the phenotyping study. The unrecovered group had moderate to severe illness as indicated by Patient-Reported Outcomes.

Measurement Information System-29 [26] score of 45 or lower on the physical sub-score, fatigue that does not resolve with rest and one additional symptom from the Centers for Disease Control and Prevention (CDC) SI screener. Individuals were excluded from the study if they had medical or psychiatric conditions diagnosed before testing positive for SARS-CoV-2. Examples of exclusions were: severe chronic obstructive pulmonary disease, organ failure, chronic infection, rheumatic and chronic inflammatory disease, chronic lung disease, or major neurologic disease. In addition, the following were assessed during clinical visits and patients were excluded if there was evidence of abnormal diastolic function or cardiomyopathy and/or O_2_ saturation of 92% or below on the 6 min exercise. Serum from healthy subjects (*n* = 13, 6 females and 7 males, mean age was 57 years old) was purchased from BioIvt Elevating Science (Hicksville, NY).

### 2.2 Human microglia cell culture

The immortalized human microglia-SV40 cell line (hTERT; T0251) derived from primary human microglia was purchased from Applied Biological Materials Inc. (ABM Inc.; Richmond, BC, Canada) and cultured in Prigrow III medium supplemented with 10% fetal bovine serum and 1% penicillin/streptomycin in type I collagen-coated T25-flasks (BD PureCoat™ ECM Mimetic Cultureware Collagen I peptide plates, Becton Dickinson, Bedford, MA) as recommended by the supplier. Microglia-SV40 maintains their phenotype and proliferation rates for about ten passages, during which all experiments were carried out using multiple thaws and sub-cultured cells. Experiments were carried out in type I collagen-coated plates (Becton Dickinson). Cell viability was determined by trypan blue (0.4%) exclusion.

### 2.3 Cell treatments

SV40 microglia (2.5 × 10^5^ cells) were stimulated with recombinant full-length SARS-CoV-2 Spike protein (Abcam, Waltham, MA, USA) at 10 ng/mL or lipopolysaccharide (LPS) and neurotensin (NT) (from Sigma-Aldrich, St. Paul, MN, USA) at 10 ng/mL and 10 nM, respectively, for 24 h. MMP-9 was measured in the supernatant fluid by enzyme-linked immunosorbent assay (ELISA) (BioTechne, Minneapolis, MN) kits according to the manufacturer’s instructions. Control cells were treated with an equal volume of culture medium in all experiments. We pre-incubated microglia with either luteolin or methlut (50 μM from CAS Biosciences, NY, USA) in dimethylsulfoxide (<1% final concentration) for 10 min before incubation with Spike protein for 24 h and then measured MMP-9 in the cell culture supernatant by ELISA.

### 2.4 Statistical analysis

All experimental conditions were performed in triplicate and all experiments were repeated at least three times (*n* = 3). Results from cultured cells are presented as mean ± SD. Comparisons between control and stimulated cells were performed using either parametric tests (unpaired 2-tailed, Student’s *t*-test, for independent samples) or Mann–Whitney non-parametric test depending on the normality of distribution that was checked with the Shapiro–Wilk’s test. Comparisons among groups were performed with one-way analysis of variance (ANOVA) followed by *post-hoc* analysis by Dunnett’s Multiple Comparison Test or the Wilcoxon *post-hoc* paired rank sum test. All statistical analyses were performed by using GraphPad Prism version 10.0.3 (275) (GraphPad Software, Boston, MA, USA).


**Ethical statement**: The research related to human use has been complied with all the relevant national regulations, institutional policies and in accordance the tenets of the Helsinki Declaration, and has been approved by the authors’ institutional review board or equivalent committee; and the specific national laws have been observed.
**Informed consent**: Informed consent has been obtained from all individuals included in this study.

## 3 Results

**Figure 1:**
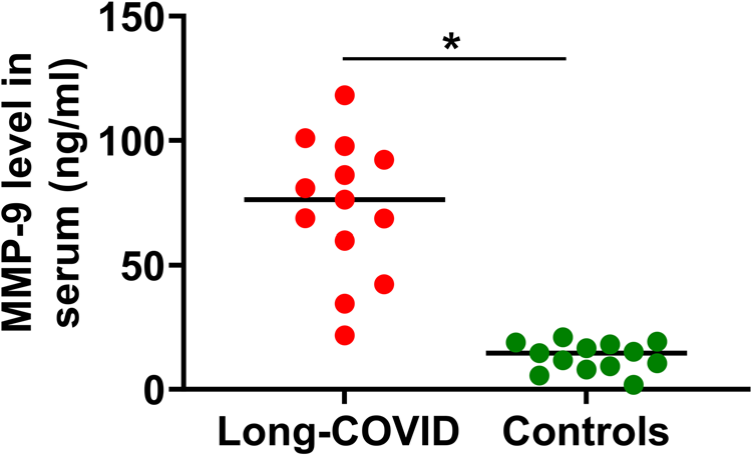
Scattergram of serum values of MMP-9. MMP-9 was measured in the serum of long COVID patients (*n* = 13, mean age 57 years) and healthy control subjects (*n* = 13, mean age 57 years) using commercial ELISA kit. *p < 0.05; *t*-test compared to control subjects.

We first measured serum MMP-9. There were significantly increased levels of MMP-9 in the serum of long COVID patients compared to healthy control subjects (Figure 1).

**Figure 2:**
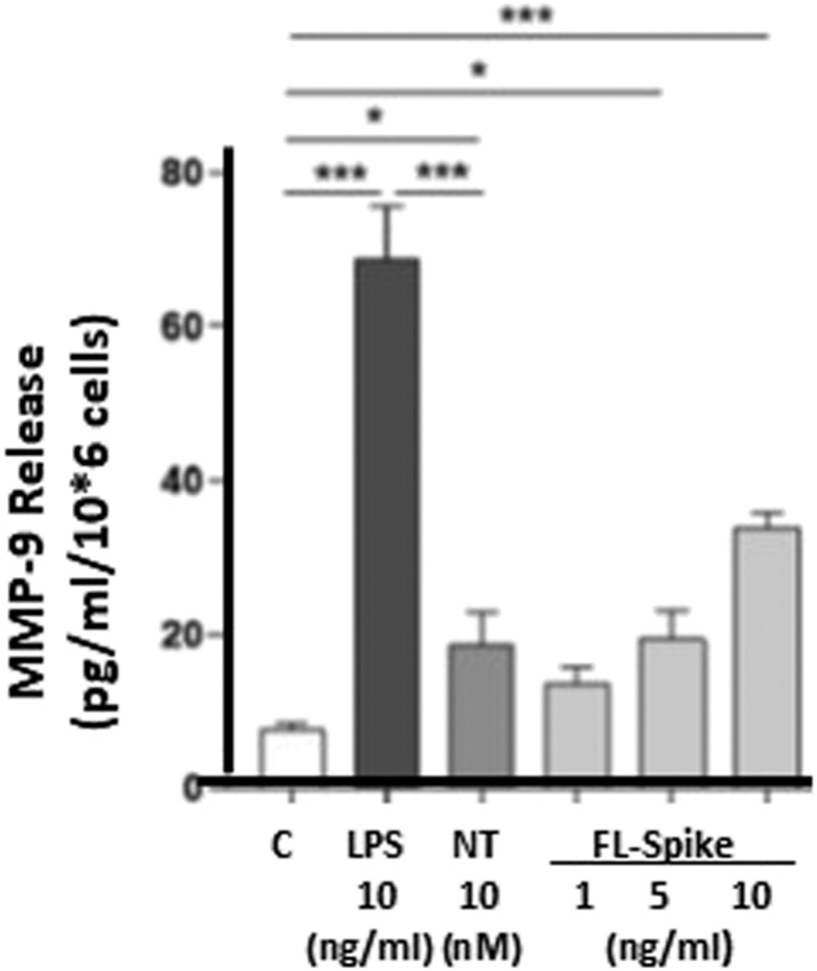
MMP-9 release from human microglia stimulated by SARS-CoV-2 Spike protein. SV-40 microglia (2.5 × 10^5^ cells) were stimulated with recombinant full-length SARS-CoV-2 Spike protein (FL-Spike, 1, 5, 10 ng/mL), LPS (10 ng/mL), or NT (10 nM) for 24 h, and MMP-9 was measured in the supernatant fluid by ELISA. LPS and NT were used as “positive triggers.” C = control. All conditions were performed in triplicate for each dataset and repeated three times (*n* = 3). Results are presented as mean ± standard error of the mean (SEM). Statistical significance is indicated as *p < 0.05 and ***p < 0.001.

We then investigated whether SARS-CoV-2 Spike protein could stimulate the release of MMP-9 from cultured human SV-40 microglia cell line. Incubation with the Spike protein (1, 5, and 10 ng/mL) for 24 h significantly increased the release of MMP-9 from microglia (Figure 2). LPS and NT used as “positive” triggers also significantly increased MMP-9 release compared to unstimulated control cells (Figure 2).

**Figure 3:**
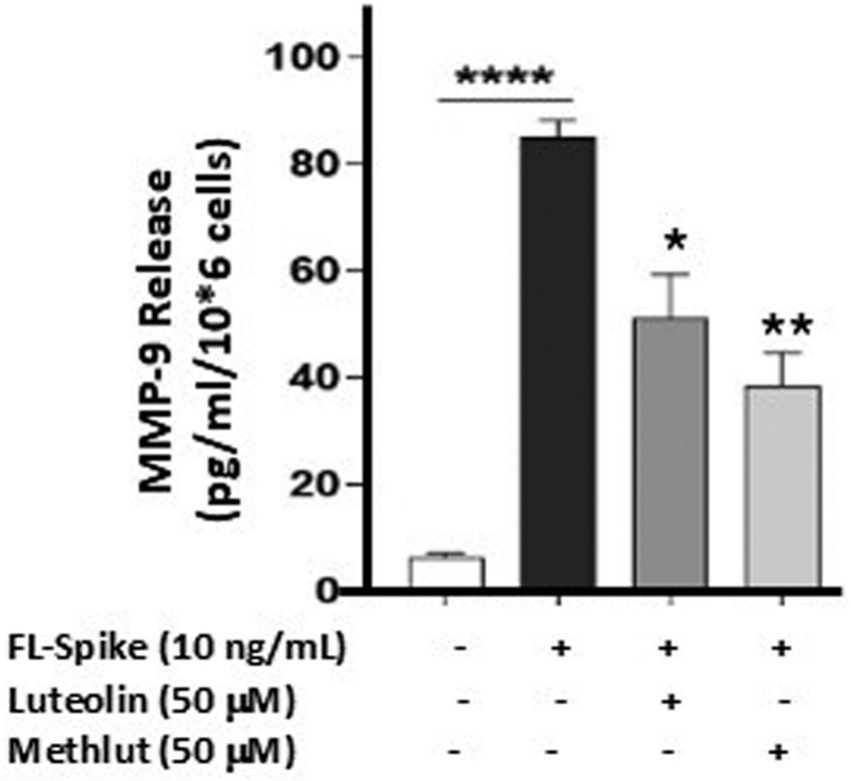
Inhibition of SARS-CoV-2 Spike protein-stimulated release of MMP-9 from human microglia by flavonoids. SV-40 microglia (2.5 × 10^5^ cells) was stimulated with recombinant full-length SARS-CoV-2 Spike protein (FL-Spike, 10 ng/mL) for 24 h after pre-incubation (10 min) with either luteolin or methlut (50 μM). MMP-9 was measured in the supernatant fluid by ELISA. All conditions were performed in triplicate for each dataset and repeated three times (*n* = 3). Results are presented as mean ± SEM. Conditions with flavonoids were compared to the one with FL-Spike protein. Statistical significance is indicated as *p < 0.05; **p < 0.01; ****p < 0.0001.

We then investigated the possible inhibitory effect of the flavonoids luteolin (3′,4′,5,7-tetrahydroxyflavone) and tetramethoxyluteolin (3′,4′,5,7-tetramethoxyflavone, methlut). Pre-incubation of the microglia with either flavonoid (50 μM) for 10 min significantly inhibited the release of MMP-9 stimulated by the Spike protein (Figure 3).

## 4 Discussion

Here we show that MMP-9 is elevated in the serum of long COVID patients. Elevated serum MMP-9 levels have been reported in COVID-19 [27] and were associated with symptom severity [24,25]. MMP-9 polymorphisms were also reported to increase the susceptibility to COVID-19, especially when accompanied by neurologic symptoms [13]. Blood MMP-9 levels were also reported to be higher in the acute phase of Neuro COVID patients [13]. MMP-9 has been associated with reduced BBB integrity [13,18].

We also show that microglia release MMP-9 when stimulated by SARS-CoV-2 Spike protein. We had previously reported that SARS-CoV-2 Spike protein stimulated cultured human microglia to secrete IL-1β, IL-18, and S100β, associated with brain damage [28]. Additional evidence indicates that the Spike protein can directly activate microglia [29–31] leading to proinflammatory effects.

SARS-CoV-2 has not been shown to infect brain cells [32–34]. The neurological issues of long COVID may, therefore, be attributed to the SARS-CoV-2 Spike protein [35] stimulating microglia [36,37]. Perivascular inflammation with lymphocytic and microglial infiltration was noted in the brains of 52 deceased patients with COVID-19 [38]. Microglia have been considered key players in the development of neuroinflammatory [39] and neurodegenerative disorders [40,41]. The duration of long COVID may depend on the length of antigen presence since it was reported that Spike protein was detected in CD16^+^ monocytes in long COVID patients up to 15–24 months post-infection [42] and inside extracellular vesicles for up to 1 year [43,44]. Recent papers reported that the SARS-CoV-2 Spike protein could be detected in long COVID patients for 6–12 months [45]. One study reported the presence of antibodies against Spike protein in the cerebrospinal fluid of 12 patients with COVID-19 [46], as well as its presence in “reservoirs” [47] including the “skull–meninges–brain axis” [48].

Our results also show that the flavonoids luteolin and methlut could inhibit the release of MMP-9 stimulated by the Spike protein. We had previously shown that these flavonoids could inhibit cultured human microglia stimulated by NT to release IL-1β [49] and reduce “brain fog” associated with long COVID [50]. In particular, nobiletin (hexamethoxyflavone) was reported to inhibit MMP-9 [51]. Another paper reported that methoxylated flavones inhibited tumor necrosis factor-mediated induction of MMP-9 [52].

Others have reported that polyphenolic compounds can lower MMP-9 levels *in vivo* and *in vitro* [53–55]. In particular, resveratrol prevented ischemic brain injury in a mouse model by inhibiting the activation of proinflammatory microglia via the MMP-9 pathway [56]. Interestingly, minocycline has been reported to reduce MMP-9 effects in rodents [57–59]. Statins have also been reported to have a beneficial effect by reducing hippocampal MMP-9 levels in a rat model of cognitive decline [60] and patients with acute ischemic stroke [61]. In fact, MMP-9 inhibitors have been considered for the treatment of traumatic brain injury [62].

There are limitations in this study: (a) we do not know the original severity of COVID-19 in the patients studied, (b) the results were compared to healthy controls and not recovered long COVID patients, and (c) the source of MMP-9 in the serum of long COVID patients is also not exactly known since SARS-CoV-2 could also stimulate the release of MMP-9 from cultured macrophages [63] and several cell types in the brain [64]. Another paper reported that cultured human mast cells can also produce MMP-9 [65].

We believe MMP-9 from Spike protein-stimulated microglia could contribute to the development of long COVID and may serve as a target for treatment including the use of luteolin, especially in a liposomal form in olive pomace oil to increase absorption (PureLut^R^).

## References

[1] Phillips S, Williams MA. Confronting our next national health disaster – long-haul covid. N Engl J Med. 2021;385(7):577–9. 10.1056/NEJMp2109285 34192429

[2] Thaweethai T, Jolley SE, Karlson EW, Levitan EB, Levy B, McComsey GA, et al. Development of a definition of postacute sequelae of SARS-CoV-2 infection. JAMA. 2023;329(22):1934–46. 10.1001/jama.2023.8823 PMC10214179 37278994

[3] Farias L, Saboya MF, Ponte Fernandes N, Perdigao Neto LV, de Arruda EAG. Development of H51Y and E157Q mutations for integrase inhibitor resistance in a patient undergoing treatment for pulmonary tuberculosis: a case report. SAGE Open Med Case Rep. 2023;11:2050313X231220786. 10.1177/2050313X231220786 PMC10752045 38152686

[4] Ali ST, Kang AK, Patel TR, Clark JR, Perez-Giraldo GS, Orban ZS, et al. Evolution of neurologic symptoms in non-hospitalized COVID-19 “long haulers”. Ann Clin Transl Neurol. 2022;9(7):950–61. 10.1002/acn3.51570 PMC9268866 35607826

[5] Almulla AF, Al-Hakeim HK. Editorial: Neuropsychiatric and neurodegenerative aspects of acute and long COVID. Front Mol Neurosci. 2023;16:1343930. 10.3389/fnmol.2023.1343930 PMC10751905 38152586

[6] Shanley JE, Valenciano AF, Timmons G, Miner AE, Kakarla V, Rempe T, et al. Longitudinal evaluation of neurologic-post acute sequelae SARS-CoV-2 infection symptoms. Ann Clin Transl Neurol. 2022;9(7):995–1010. 10.1002/acn3.51578 PMC9268882 35702954

[7] Meshkat S, Salimi A, Joshaghanian A, Sedighi S, Sedighi S, Aghamollaii V. Chronic neurological diseases and COVID-19: associations and considerations. Transl Neurosci. 2020;11(1):294–301. 10.1515/tnsci-2020-0141 PMC7712023 33335769

[8] Proal AD, VanElzakker MB. Long COVID or post-acute sequelae of COVID-19 (PASC): an overview of biological factors that may contribute to persistent symptoms. Front Microbiol. 2021;12:698169. 10.3389/fmicb.2021.698169 PMC8260991 34248921

[9] Meinhardt J, Radke J, Dittmayer C, Franz J, Thomas C, Mothes R, et al. Olfactory transmucosal SARS-CoV-2 invasion as a port of central nervous system entry in individuals with COVID-19. Nat Neurosci. 2021;24(2):168–75. 10.1038/s41593-020-00758-5 33257876

[10] Sodagar A, Javed R, Tahir H, Razak SIA, Shakir M, Naeem M, et al. Pathological features and neuroinflammatory mechanisms of SARS-CoV-2 in the brain and potential therapeutic approaches. Biomolecules. 2022;12(7):971. 10.3390/biom12070971 PMC9313047 35883527

[11] Tremblay ME, Madore C, Tian L, Verkhratsky A. Editorial: Role of neuroinflammation in the neuropsychiatric and neurological aspects of COVID-19. Front Cell Neurosci. 2022;16:840121. 10.3389/fncel.2022.840121 PMC8863733 35221927

[12] Lee MH, Perl DP, Nair G, Li W, Maric D, Murray H, et al. Microvascular injury in the brains of patients with covid-19. N Engl J Med. 2021;384(5):481–3. 10.1056/NEJMc2033369 PMC7787217 33378608

[13] Bonetto V, Pasetto L, Lisi I, Carbonara M, Zangari R, Ferrari E, et al. Markers of blood–brain barrier disruption increase early and persistently in COVID-19 patients with neurological manifestations. Front Immunol. 2022;13:1070379. 10.3389/fimmu.2022.1070379 PMC9798841 36591311

[14] Zingaropoli MA, Iannetta M, Piermatteo L, Pasculli P, Latronico T, Mazzuti L, et al. Neuro-axonal damage and alteration of blood-brain barrier integrity in COVID-19 patients. Cells. 2022;11(16):2480. 10.3390/cells11162480 PMC9406414 36010557

[15] Yang AC, Kern F, Losada PM, Agam MR, Maat CA, Schmartz GP, et al. Dysregulation of brain and choroid plexus cell types in severe COVID-19. Nature. 2021;595(7868):565–71. 10.1038/s41586-021-03710-0 PMC8400927 34153974

[16] Xu Z, Wang H, Jiang S, Teng J, Zhou D, Chen Z, et al. Brain pathology in COVID-19: clinical manifestations and potential mechanisms. Neurosci Bull. 2024;40(3):383–400. 10.1007/s12264-023-01110-0 PMC10912108 37715924

[17] Gu X, Wang S, Zhang W, Li C, Guo L, Wang Z, et al. Probing long COVID through a proteomic lens: a comprehensive two-year longitudinal cohort study of hospitalised survivors. EBioMedicine. 2023;98:104851. 10.1016/j.ebiom.2023.104851 PMC10660018 37924708

[18] Rempe RG, Hartz AM, Bauer B. Matrix metalloproteinases in the brain and blood-brain barrier: versatile breakers and makers. J Cereb Blood Flow Metab. 2016;36(9):1481–507. 10.1177/0271678X16655551 PMC5012524 27323783

[19] Lopez-Navarro ER, Gutierrez J. Metalloproteinases and their inhibitors in neurological disease. Naunyn Schmiedebergs Arch Pharmacol. 2022;395(1):27–38. 10.1007/s00210-021-02188-x 34851449

[20] Kaczmarek KT, Protokowicz K, Kaczmarek L. Matrix metalloproteinase-9: a magic drug target in neuropsychiatry? J Neurochem. 2023. 10.1111/jnc.15976 37791997

[21] Beroun A, Mitra S, Michaluk P, Pijet B, Stefaniuk M, Kaczmarek L. MMPs in learning and memory and neuropsychiatric disorders. Cell Mol Life Sci. 2019;76(16):3207–28. 10.1007/s00018-019-03180-8 PMC6647627 31172215

[22] Stawarski M, Stefaniuk M, Wlodarczyk J. Matrix metalloproteinase-9 involvement in the structural plasticity of dendritic spines. Front Neuroanat. 2014;8:68. 10.3389/fnana.2014.00068 PMC4091410 25071472

[23] Dhanda S, Sandhir R. Blood–brain barrier permeability is exacerbated in experimental model of hepatic encephalopathy via MMP-9 activation and downregulation of tight junction proteins. Mol Neurobiol. 2018;55(5):3642–59. 10.1007/s12035-017-0521-7 28523565

[24] Ding L, Guo H, Zhang C, Jin H, Guo X, Li T. Elevated matrix metalloproteinase‑9 expression is associated with COVID‑19 severity: a meta‑analysis. Exp Ther Med. 2023;26(6):545. 10.3892/etm.2023.12244 PMC10623216 37928509

[25] Savic G, Stevanovic I, Mihajlovic D, Jurisevic M, Gajovic N, Jovanovic I, et al. MMP-9/BDNF ratio predicts more severe COVID-19 outcomes. Int J Med Sci. 2022;19(13):1903–11. 10.7150/ijms.75337 PMC9682503 36438922

[26] Bonnell LN, Clifton J, Natkin LW, Hitt JR, Littenberg B. The association of self-perceived changes due to COVID-19 with mental and physical health among adult primary care patients with multiple chronic conditions: a US-based longitudinal study. J Multimorb Comorb. 2024;14:26335565231222148. 10.1177/26335565231222148 PMC10798126 38250744

[27] Ueland T, Holter JC, Holten AR, Muller KE, Lind A, Bekken GK, et al. Distinct and early increase in circulating MMP-9 in COVID-19 patients with respiratory failure. J Infect. 2020;81(3):e41–3. 10.1016/j.jinf.2020.06.061 PMC7320854 32603675

[28] Tsilioni I, Theoharides TC. Recombinant SARS-CoV-2 spike protein and its receptor binding domain stimulate release of different pro-inflammatory mediators via activation of distinct receptors on human microglia cells. Mol Neurobiol. 2023;60(11):6704–14. 10.1007/s12035-023-03493-7 37477768

[29] Jeong GU, Lyu J, Kim KD, Chung YC, Yoon GY, Lee S, et al. SARS-CoV-2 infection of microglia elicits proinflammatory activation and apoptotic cell death. Microbiol Spectr. 2022;10(3):e0109122. 10.1128/spectrum.01091-22 PMC9241873 35510852

[30] Olajide OA, Iwuanyanwu VU, Adegbola OD, Al-Hindawi AA. SARS-CoV-2 spike glycoprotein S1 induces neuroinflammation in BV-2 microglia. Mol Neurobiol. 2022;59(1):445–58. 10.1007/s12035-021-02593-6 PMC8551352 34709564

[31] Samudyata N, Oliveira AO, Malwade S, Rufino de Sousa N, Goparaju SK, Gracias J, et al. SARS-CoV-2 promotes microglial synapse elimination in human brain organoids. Mol Psychiatry. 2022;27:3939–50. 10.1038/s41380-022-01786-2 PMC9533278 36198765

[32] Placantonakis DG, Aguero-Rosenfeld M, Flaifel A, Colavito J, Inglima K, Zagzag D, et al. SARS-CoV-2 is not detected in the cerebrospinal fluid of encephalopathic COVID-19 patients. Front Neurol. 2020;11:587384. 10.3389/fneur.2020.587384 PMC7759491 33362695

[33] Thakur KT, Miller EH, Glendinning MD, Al-Dalahmah O, Banu MA, Boehme AK, et al. COVID-19 neuropathology at Columbia University Irving Medical Center/New York Presbyterian Hospital. Brain. 2021;144(9):2696–708. 10.1093/brain/awab148 PMC8083258 33856027

[34] Lewis A, Frontera J, Placantonakis DG, Galetta S, Balcer L, Melmed KR. Cerebrospinal fluid from COVID-19 patients with olfactory/gustatory dysfunction: a review. Clin Neurol Neurosurg. 2021;207:106760. 10.1016/j.clineuro.2021.106760 PMC8196517 34146842

[35] Theoharides TC, Kempuraj D. Role of SARS-CoV-2 spike-protein-induced activation of microglia and mast cells in the pathogenesis of neuro-COVID. Cells. 2023;12(5):688. 10.3390/cells12050688 PMC10001285 36899824

[36] Matschke J, Lutgehetmann M, Hagel C, Sperhake JP, Schroder AS, Edler C, et al. Neuropathology of patients with COVID-19 in Germany: a post-mortem case series. Lancet Neurol. 2020;19(11):919–29. 10.1016/S1474-4422(20)30308-2 PMC7535629 33031735

[37] Dey R, Bishayi B. Microglial inflammatory responses to SARS-CoV-2 infection: a comprehensive review. Cell Mol Neurobiol. 2023;44(1):2. 10.1007/s10571-023-01444-3 PMC11407175 38099973

[38] Wierzba-Bobrowicz T, Krajewski P, Tarka S, Acewicz A, Felczak P, Stępień T, et al. Neuropathological analysis of the brains of fifty-two patients with COVID-19. Folia Neuropathol. 2021;59(3):219–31. 10.5114/fn.2021.108829 34628787

[39] Bachiller S, Jimenez-Ferrer I, Paulus A, Yang Y, Swanberg M, Deierborg T, et al. Microglia in neurological diseases: a road map to brain-disease dependent-inflammatory response. Front Cell Neurosci. 2018;12:488. 10.3389/fncel.2018.00488 PMC6305407 30618635

[40] Perry VH, Nicoll JA, Holmes C. Microglia in neurodegenerative disease. Nat Rev Neurol. 2010;6(4):193–201. 10.1038/nrneurol.2010.17 20234358

[41] Hickman S, Izzy S, Sen P, Morsett L, El KJ. Microglia in neurodegeneration. NatNeurosci. 2018;21(10):1359–69. 10.1038/s41593-018-0242-x PMC6817969 30258234

[42] Patterson BK, Francisco EB, Yogendra R, Long E, Pise A, Rodrigues H, et al. Persistence of SARS CoV-2 S1 protein in CD16+ monocytes in post-acute sequelae of COVID-19 (PASC) up to 15 months post-infection. Front Immunol. 2021;12:746021. 10.3389/fimmu.2021.746021 PMC8784688 35082777

[43] Craddock V, Mahajan A, Spikes L, Krishnamachary B, Ram AK, Kumar A, et al. Persistent circulation of soluble and extracellular vesicle-linked Spike protein in individuals with postacute sequelae of COVID-19. J Med Virol. 2023;95(2):e28568. 10.1002/jmv.28568 PMC10048846 36756925

[44] Peluso MJ, Deeks SG, Mustapic M, Kapogiannis D, Henrich TJ, Lu S, et al. SARS-CoV-2 and mitochondrial proteins in neural-derived exosomes of COVID-19. Ann Neurol. 2022;91(6):772–81. 10.1002/ana.26350 PMC9082480 35285072

[45] Swank Z, Senussi Y, Manickas-Hill Z, Yu XG, Li JZ, Alter G, et al. Persistent circulating severe acute respiratory syndrome coronavirus 2 spike is associated with post-acute coronavirus disease 2019 sequelae. Clin Infect Dis. 2023;76(3):e487–90. 10.1093/cid/ciac722 PMC10169416 36052466

[46] Lewis A, Frontera J, Placantonakis DG, Lighter J, Galetta S, Balcer L, et al. Cerebrospinal fluid in COVID-19: a systematic review of the literature. J Neurol Sci. 2021;421:117316. 10.1016/j.jns.2021.117316 PMC7833669 33561753

[47] Proal AD, VanElzakker MB, Aleman S, Bach K, Boribong BP, Buggert M, et al. Author correction: SARS-CoV-2 reservoir in post-acute sequelae of COVID-19 (PASC). Nat Immunol. 2023;24(10):1778. 10.1038/s41590-023-01646-3 37723351

[48] Rong Z, Mai H, Kapoor S, Puelles VG, Czogalla J, Schädler J, et al. SARS-CoV-2 spike protein accumulation in the skull-meninges brain axis: potential implications for long-term neurological complications in post-COVID-19. bioRxiv. 2023. 10.1101/2023.04.04.535604

[49] Patel AB, Tsilioni I, Leeman SE, Theoharides TC. Neurotensin stimulates sortilin and mTOR in human microglia inhibitable by methoxyluteolin, a potential therapeutic target for autism. Proc Natl Acad Sci U S A. 2016;113(45):E7049–58. 10.1073/pnas.1604992113 PMC5111711 27663735

[50] Theoharides TC, Cholevas C, Polyzoidis K, Politis A. Long-COVID syndrome-associated brain fog and chemofog: Luteolin to the rescue. Biofactors. 2021;47(2):232–41. 10.1002/biof.1726 PMC8250989 33847020

[51] Kim JJ, Korm S, Kim WS, Kim OS, Lee JS, Min HG, et al. Nobiletin suppresses MMP-9 expression through modulation of p38 MAPK activity in human dermal fibrobalsts. Biol Pharm Bull. 2014;37(1):158–63. 10.1248/bpb.b13-00534 24389490

[52] Kamiya T, Mizuno N, Hayashi K, Otsuka T, Haba M, Abe N, et al. Methoxylated flavones from Casimiroa edulis La Llave suppress MMP9 expression via inhibition of the JAK/STAT3 pathway and TNF alpha-dependent pathways. J Agric Food Chem. 2024;72(26):14678–83. 10.1021/acs.jafc.4c00965 38910321

[53] Pagliara V, De Rosa M, Di Donato P, Nasso R, D’Errico A, Cammarota F, et al. Inhibition of interleukin-6-induced matrix metalloproteinase-2 expression and invasive ability of lemon peel polyphenol extract in human primary colon cancer cells. Molecules. 2021;26(23):7076. 10.3390/molecules26237076 PMC8658805 34885656

[54] Milton-Laskibar I, Trepiana J, Macarulla MT, Gomez-Zorita S, Arellano-Garcia L, Fernandez-Quintela A, et al. Potential usefulness of mediterranean diet polyphenols against COVID-19-induced inflammation: a review of the current knowledge. J Physiol Biochem. 2023;79(2):371–82. 10.1007/s13105-022-00926-0 PMC9641689 36346507

[55] Islam MT, Jang NH, Lee HJ. Natural products as regulators against matrix metalloproteinases for the treatment of cancer. Biomedicines. 2024;12(4):794. 10.3390/biomedicines12040794 PMC11048580 38672151

[56] Zhang H, Zhao W. Resveratrol alleviates ischemic brain injury by inhibiting the activation of pro-inflammatory microglia via the CD147/MMP-9 pathway. J Stroke Cerebrovasc Dis. 2022;31(4):106307. 10.1016/j.jstrokecerebrovasdis.2022.106307 35093629

[57] Hahn JN, Kaushik DK, Mishra MK, Wang J, Silva C, Yong VW. Impact of minocycline on extracellular matrix metalloproteinase inducer, a factor implicated in multiple sclerosis immunopathogenesis. J Immunol. 2016;197(10):3850–60. 10.4049/jimmunol.1600436 27733550

[58] Toledo MA, Wen TH, Binder DK, Ethell IM, Razak KA. Reversal of ultrasonic vocalization deficits in a mouse model of Fragile X Syndrome with minocycline treatment or genetic reduction of MMP-9. Behav Brain Res. 2019;372:112068. 10.1016/j.bbr.2019.112068 PMC6662633 31271818

[59] Tras B, Eser Faki H, Ozdemir Kutahya Z, Bahcivan E, Dik B, Uney K. The effects of dexamethasone and minocycline alone and combined with N-acetylcysteine and vitamin E on serum matrix metalloproteinase-9 and coenzyme Q10 levels in aflatoxin B1 administered rats. Pol J Vet Sci. 2022;25(3):419–27. 10.24425/pjvs.2022.142026 36156107

[60] Adeli S, Zahmatkesh M, Ansari Dezfouli M. Simvastatin attenuates hippocampal MMP-9 expression in the streptozotocin-induced cognitive impairment. Iran Biomed J. 2019;23(4):262–71. 10.29252/.23.4.262 PMC6462290 30218997

[61] Naji MT, Al-Kuraishy HM, Al-Gareeb AI. Differential effects of statins on matrix metalloproteinase-9 (MMP-9) in patients with acute ischaemic stroke: a potential for salutary. J Pak Med Assoc. 2021;71(12):S82–7. 35130225

[62] Sunny A, James RR, Menon SR, Rayaroth S, Daniel A, Thompson NA, et al. Matrix Metalloproteinase-9 inhibitors as therapeutic drugs for traumatic brain injury. Neurochem Int. 2024;172:105642. 10.1016/j.neuint.2023.105642 38008261

[63] Murphy SL, Halvorsen B, Holter JC, Huse C, Tveita A, Troseid M, et al. Circulating markers of extracellular matrix remodelling in severe COVID-19 patients. J Intern Med. 2023;294(6):784–97. 10.1111/joim.13725 37718572

[64] Vafadari B, Salamian A, Kaczmarek L. MMP-9 in translation: from molecule to brain physiology, pathology, and therapy. J Neurochem. 2016;139(Suppl 2):91–114. 10.1111/jnc.13415 26525923

[65] Kimata M, Ishizaki M, Tanaka H, Nagai H, Inagaki N. Production of matrix metalloproteinases in human cultured mast cells: involvement of protein kinase C-mitogen activated protein kinase kinase-extracellular signal-regulated kinase pathway. Allergol Int. 2006;55(1):67–76. 10.2332/allergolint.55.67 17075289

